# Supplementary material for: The Evolution and Characterization of the RNA Interference Pathways in Lophotrochozoa
Source: Genome Biol Evol. 2024 May 7;16(5):evae098. doi: 10.1093/gbe/evae098 (PMC11114477; doi:10.1093/gbe/evae098)
Supplement: evae098_Supplementary_Data [file evae098_supplementary_data.zip › Supplementary_materials.zip!/selected_miRNAs/NoGe_selected_miRNAs/known_miRNA_families_pdf_files/NMRB01000014.1_Notospermus_geniculatus_strain_Ushimado_scaffold14,_whole_genome_shotgun_sequence_617.pdf]

Provisional ID	:	NMRB01000014.1_Notospermus_geniculatus_strain_Ushimado_scaffold14,_whole_genome_shotgun_sequence_617
Score total	:	977762.4
Score for star read(s)	:	3.9
Score for read counts	:	977755.5
Score for mfe	:	0
Score for randfold	:	0
Score for cons. seed	:	3
Total read count	:	1917837
Mature read count	:	1917797
Loop read count	:	0
Star read count	:	40

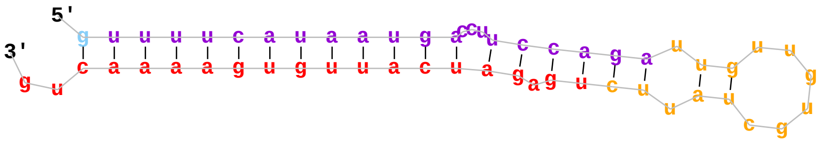

Genome coordinates: NMRB01000014.1 432426-432481

pre-miRNA sequence:
UUUUCAUAAUGACCUUCCAGAUUUUCAUAAUGACCUUCCAGAUUGUUGUGCUAUUCUGAGAUCAUUGUGAAAACUG

Mature sequence: UGAGAUCAUUGUGAAAACUG

Aligned mature sequence of the *N. geniculatus* pre-miRNA with the top 5 hits from the BLAST search against the pre-miRNA mirGeneDB set:

```
                               **************** **
Nge_Bantam-P16                 ugagaucauugugaaaacug---
Lan-Bantam_3p                  ugagaucauugugaaaacugauu
Cgi-Bantam_3p                  ugagaucauugugaaaacugauu
Cte-Bantam_3p                  ugagaucauugugaaaacuaauc
Ovu-Bantam_3p                  ugagaucauugugaaaacugguu
Csc-Bantam-P16_3p              ugagaucauugugaaagcugauu
```
